# An Open Environment CT-US Fusion for Tissue Segmentation during Interventional Guidance

**DOI:** 10.1371/journal.pone.0027372

**Published:** 2011-11-23

**Authors:** Charles F. Caskey, Mario Hlawitschka, Shengping Qin, Lisa M. Mahakian, Robert D. Cardiff, John M. Boone, Katherine W. Ferrara

**Affiliations:** 1 Department of Biomedical Engineering, University of California Davis, Davis, California, United States of America; 2 Department of Computer Science, University of California Davis, Davis, California, United States of America; 3 Department of Pathology and Laboratory Medicine, School of Medicine, University of California Davis, Davis, California, United States of America; 4 Department of Radiology, University of California Davis, Davis, California, United States of America; National Institute of Health, United States of America

## Abstract

Therapeutic ultrasound (US) can be noninvasively focused to activate drugs, ablate tumors and deliver drugs beyond the blood brain barrier. However, well-controlled guidance of US therapy requires fusion with a navigational modality, such as magnetic resonance imaging (MRI) or X-ray computed tomography (CT). Here, we developed and validated tissue characterization using a fusion between US and CT. The performance of the CT/US fusion was quantified by the calibration error, target registration error and fiducial registration error. Met-1 tumors in the fat pads of 12 female FVB mice provided a model of developing breast cancer with which to evaluate CT-based tissue segmentation. Hounsfield units (HU) within the tumor and surrounding fat pad were quantified, validated with histology and segmented for parametric analysis (fat: −300 to 0 HU, protein-rich: 1 to 300 HU, and bone: HU>300). Our open source CT/US fusion system differentiated soft tissue, bone and fat with a spatial accuracy of ∼1 mm. Region of interest (ROI) analysis of the tumor and surrounding fat pad using a 1 mm^2^ ROI resulted in mean HU of 68±44 within the tumor and −97±52 within the fat pad adjacent to the tumor (p<0.005). The tumor area measured by CT and histology was correlated (r^2^ = 0.92), while the area designated as fat decreased with increasing tumor size (r^2^ = 0.51). Analysis of CT and histology images of the tumor and surrounding fat pad revealed an average percentage of fat of 65.3% vs. 75.2%, 36.5% vs. 48.4%, and 31.6% vs. 38.5% for tumors <75 mm^3^, 75–150 mm^3^ and >150 mm^3^, respectively. Further, CT mapped bone-soft tissue interfaces near the acoustic beam during real-time imaging. Combined CT/US is a feasible method for guiding interventions by tracking the acoustic focus within a pre-acquired CT image volume and characterizing tissues proximal to and surrounding the acoustic focus.

## Introduction

CT has long been applied for the characterization of tissues, such as fat and bone, in diagnostic imaging [1]. More recently, the use of CT in the interventional and intra-operative setting is expanding, due to advantages in speed and convenience as compared to MRI. C-arm cone-beam CT scanners with flat-panel detectors are increasingly used in interventional radiology suites for mapping and navigational applications [2]. Fusions between CT and US have been developed for the purposes of guiding biopsy and radio-frequency ablation [3], [4]. However, CT has not yet been applied to guide ultrasound therapy planning and US-based thermometry for mild hyperthermia or ultrasonic ablation.

Mild hyperthermia is an emerging technique for image-guided interventions since tumor oxygenation, vascular permeability and blood flow can be enhanced, potentially increasing the efficacy of radiotherapy and chemotherapeutic drugs [5], [6], [7], [8], [9] and activating temperature-sensitive drugs [10], [11], [12], [13]. Ultrasound is an ideal method for noninvasively generating hyperthermia due to the low cost, flexibility and potential to image and detect temperature changes in real time [14]. While MR-guided focused ultrasound can similarly monitor treatment and temperature [15], US guidance of interventions remains widespread.

In the guidance of mild hyperthermia, tissue characterization is important since the reflection of sound waves by bone can create unanticipated regions of thermal damage. Also, during thermal therapy, changes in the speed of sound produce an apparent shift in the position of tissue within and distal to the acoustic focus over successive image acquisitions, providing a basis for ultrasonic thermometry [13]. While sound speed increases with increasing temperature in non-fatty soft tissues over a temperature range of 30-50°C, sound speed decreases as temperature increases over this range in fatty tissue [16]. Therefore, ultrasound thermometry requires accurate estimation of fat content. The local temperature change is then estimated from the product of the apparent echo time shift and the tissue-dependent coefficient for thermal expansion. By estimating the percentage of fat and other tissue components and incorporating the relevant thermal expansion coefficients within each small region prior to thermal therapy, temperature changes can be mapped from shifts in ultrasound echoes [14].

Open source software environments are emerging as an important component of multi-modality imaging; for example, 3D Slicer facilitates image segmentation and OpenIGTLink is an open-source protocol for rapid transfer of generic data between software and devices used in image-guided procedures [17] [18], [19]. We set out to leverage these platforms to develop an open source fused CT/US system by interfacing a clinical US scanner and generic clinical CT scanner, and to demonstrate the utility of such a fused system by characterizing tissues relevant to ultrasound therapy (soft tissue, bone, and fat) in a mouse breast cancer tumor model [17], [19], [20].

### Study Design

Image registration between CT and US was first quantified. Next, we characterized HU-based segmentation of fat and soft tissue by comparing fat content and tumor size (n = 12) in histology and comparable CT and US slices. We tested the feasibility of using CT to identify tissue within the acoustic beam by retrospectively fusing images from US and CT clinical scanners (n = 4). These data were supplemented by tissue characterization performed on living animals using a small animal CT scanner.

## Results

OpenIGTLink has been applied to interface a Siemens Sequoia US scanner with a cone-beam breast CT via electromagnetic (EM) positioning. The resulting open source software acquires US images in real-time, computes the 2D image slice location and transmits the image and location via OpenIGTLink within 0.1 seconds (http://code.google.com/p/ct-us-openigtlink/). By using the IGSTK driver to interface with hardware and standard NTSC video capture, the fused CT/US plug-in can easily be extended to function with optically-tracked systems and other CT and US scanners. Our combined platform includes a physical interface for small animal research, in which the animal is either imaged with both modalities within the same study or moved between imaging platforms.

### Millimeter-scale Accuracy Possible with Fused CT/US

**Figure 1:**
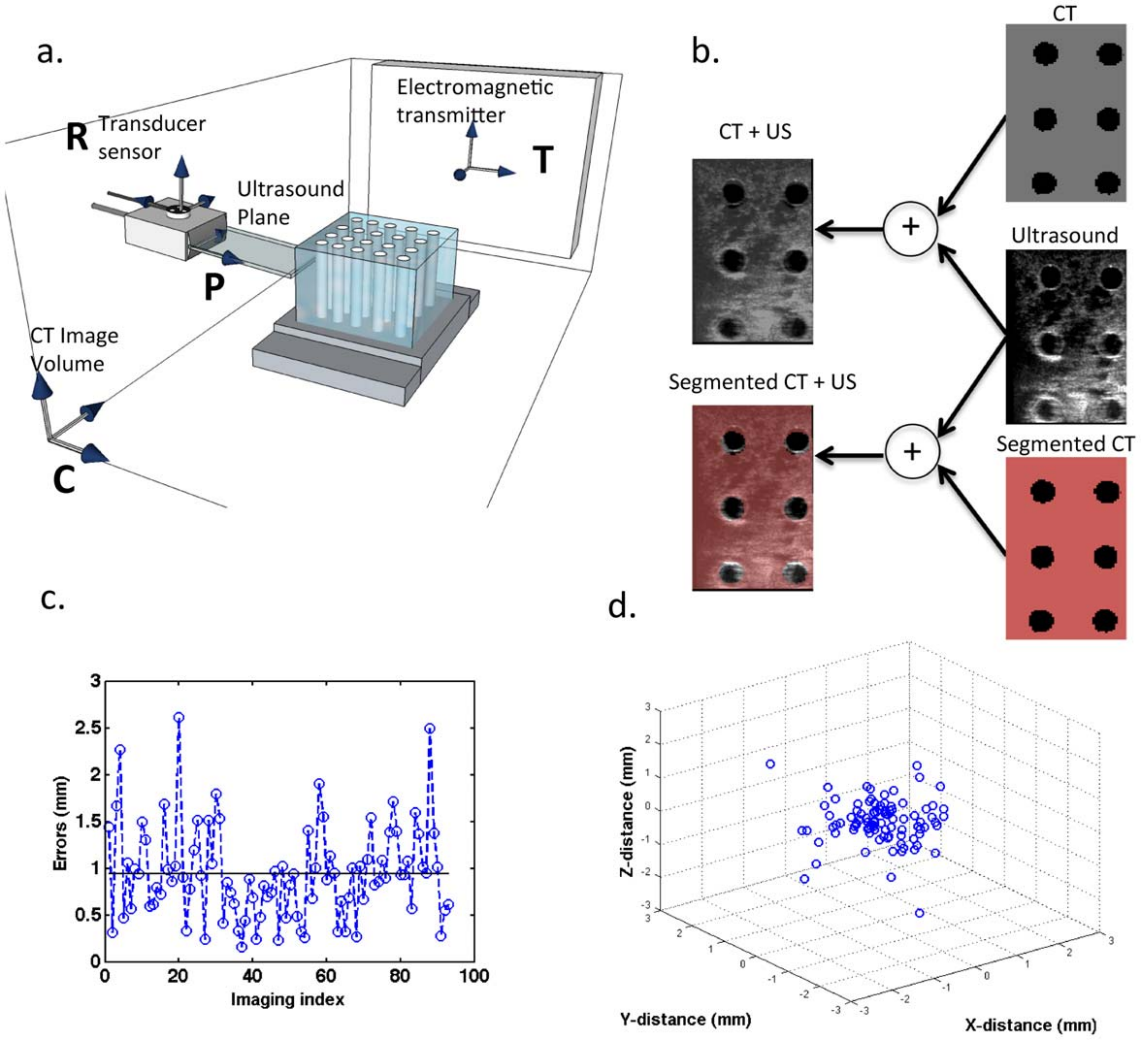
Calibration and accuracy of electromagnetically-tracked CT/US. (a) Relevant hardware for combined CT/US with the coordinate systems used to join the tracked US transducer and CT image space. The US plane, **P** space, is calibrated with respect to a position sensor, **R** space, attached to the US transducer. The sensor's location is known relative to an electromagnetic transmitter, **T** space, whose orientation relative to the CT image space, **C**, is determined by fiducial registration. (b) The combined imaging system is used to view B-mode US, the corresponding CT slice, the corresponding segmented CT slice, or any combination of these slices in real-time. (c) The magnitude of the error in calibration across 93 observations of a single point imaged from multiple angles with a mean of ∼0.94 ±0.5 mm. (d) Calibration data from (c) plotted in 3D to provide the spatial distribution of the error and indicate that the error is isotropic.

By bridging EM tracking hardware and image acquisition with 3D slicer, US images were acquired for real-time combined CT/US with mm-scale accuracy. The target registration error was indicated by the quality of the transformations between the coordinate systems described in Figure 1a. The mean error in calibration between the US plane (**P** space) and transducer sensor (**R** space) was 0.9 ± 0.5 mm. With 93 observations of a single point acquired from different angles, the maximum and minimum residual errors were 2.6 and 0.2 mm, respectively (Figure 1c, d). Registration between the CT image space, **C**, and the tracked space, **T**, was also achieved with mm-scale accuracy. The mean residual error in the transformation of twelve mutual fiducial points between **C** and **T** was 1.0 **±** 0.2 mm.

The propagation of these errors was indicated by the target registration error between the US image and comparable CT slice after co-registration of the images. Slices through the cylinders in the phantom were easily visualized in both modalities and accurate co-registration was evident by the consistent shape and alignment between the targets (Figure 1b). Circular targets in the dual modality phantom appeared as a grid of circles during combined CT/US imaging. A mean target registration error of 1.0 ± 0.3 mm was measured across 50 corresponding targets on CT and US in fused 2D images.

### Syngeneic Met-1 tumor provides model system for imaging-based tissue characterization

**Figure 2:**
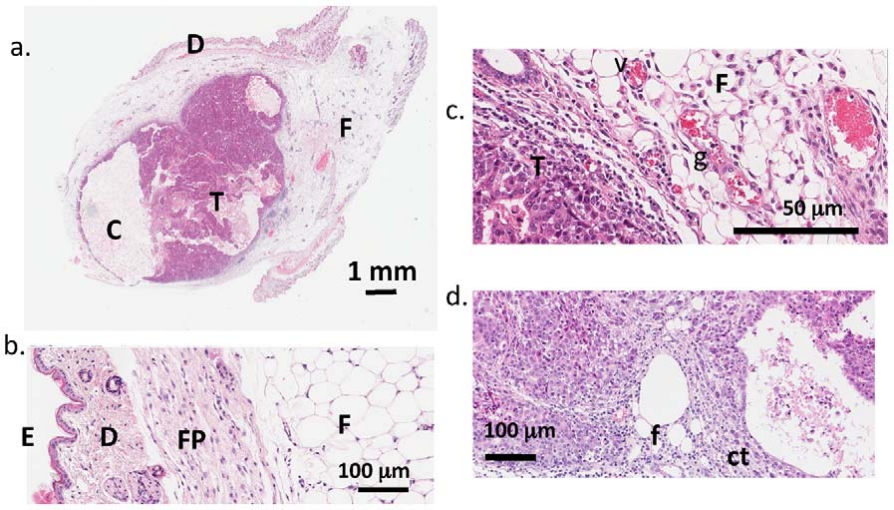
H&E stained histology of Met-1 tumors. H&E stained histology of MET-1 tumors. (a) H&E stained section of a single tumor (**T**) with multiple cysts (**C**) growing beneath the dermis (**D**) in the mammary fat pad (**F**). (b) The epidermis (**E**), dermis (**D**), fat pad fascia (**FP**) and fat (**F**) remote to the tumor has minimal inflammation. (c) In contrast, the fat pad (**F**) adjacent to the tumor (**T**) is inflamed with compressed adipocytes, dilated, engorged vessels (**V**) and scattered granulocytes (**g**). (d) The malignant nature of the tumor is illustrated by the cords of neoplastic cells infiltrating the adjacent fat (**f**) and connective tissue (**ct**).

The transplanted tumor cells formed a mass that expands within the mammary fat pad (Figure 2a). In the absence of inflammation and tumor expansion, the fat pad thickness ranged from 1 to 3 mm and extended laterally over more than 10 mm, separated from the dermal layers by the fascia (see Figure 2b). As the tumor grew within the fat pad, scattered fat cells and connective tissue were evident, while the fat cells surrounding the tumor were compressed, aspherical and disorganized (Figure 2d). Granular lymphocytes and vasodilation within the fat pad provided evidence of inflammation during tumor growth (Figure 2c).

### Tissue characterization based on echogenicity could not differentiate tumor and fat

In this study, the grayscale B-mode image amplitude was 111.0 ± 9.6, 76.8 ± 5.7, 107.8 ± 8.5, and 63.3 ± 7.9 for fat, muscle, bone and tumor, respectively. Fat and bone were differentiated from the less echogenic muscle and tumor tissue (p<0.05, multiple comparison ANOVA); however, fat and bone or muscle and tumor could not be differentiated from one another using US.

### CT successfully characterizes fat and tumor

**Figure 3:**
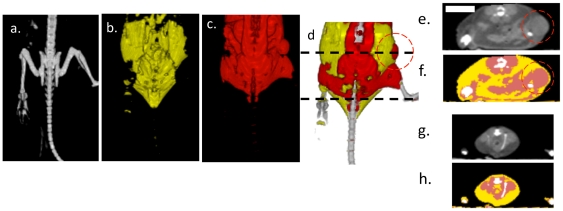
Hounsfield unit-based segmentation differentiates tissue types. (a–d) Isosurfaces of a mouse with a single hindlimb tumor, (a), bone, (b), fat, (c) soft tissue, (d), combined bone, fat and soft tissue isosurfaces. (e–h) Transverse plane CT images and segmented image from location of black dotted line in (d), where yellow indicates fat, red indicates soft tissue and white indicates bone.

Segmentation of CT images according to the HU in Table 1 indicated the location of the murine fat pads. Isosurfaces generated from segmented CT images identified bone, fat and protein-rich regions (Figure 3a–c). A fat pad on the left hind limb and a unilateral Met-1 tumor that disrupts the fat pad on the right hind limb were visible in segmented images. The fatty regions detected by CT were distributed throughout the abdomen and along the back (Figure 3d). In transverse CT images, fat pads appeared as a contiguous layer with HU in the range expected for adipose tissue (Figures 3e–h).

**Table 1:** HU Ranges for identified tissues.

HU Range	Tissue	Color
−300 to 0	Fat	Yellow
1 to 300	Protein-Rich	Pink
301 to 3000	Bone	White

The colors in the table are used throughout the paper to indicate tissue type.

**Figure 4:**
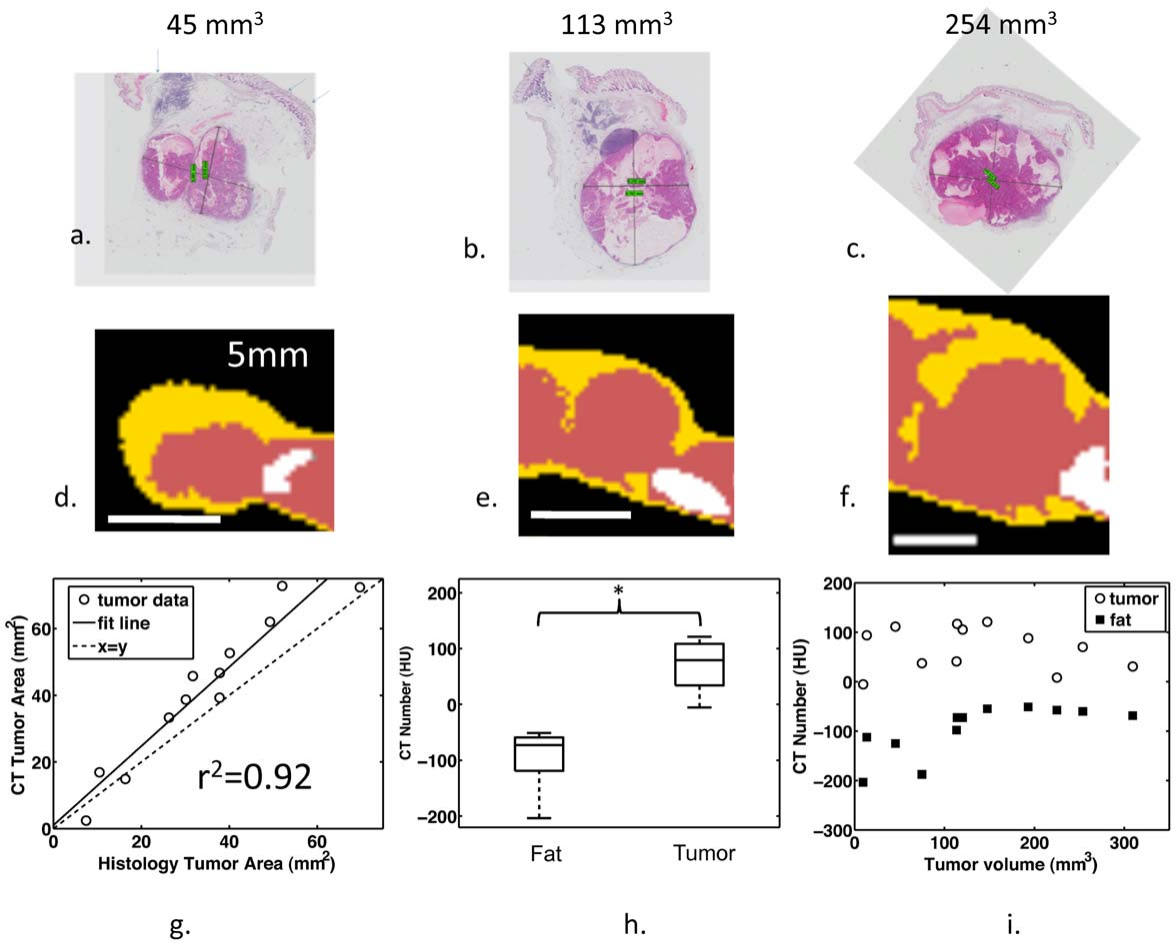
CT segmentation corresponds with histology. (a–c) Histology and (d–f) corresponding segmented CT slices for tumors of diameters 45 mm^3^, 113 mm^3^, and 254 mm^3^, respectively. (g) The tumor area in histology is correlated with the cross-sectional area of protein-rich tissue detected by CT (r^2^ = 0.92, slope = 1.2). (h) The average HU of 1 mm^2^ ROIs in the fat pad is less than within the tumor when averaged across all tumors (p<0.05). (i) The CT number is higher within the tumor compared to the surrounding fat pad for all tumors.

H&E-stained histology slides and comparable CT slices had similar morphology. Representative histology images (Figure 4a–c) and comparable CT slices (Figure 4d–f) showed a core tumor embedded in a surrounding fat pad with a thickness of 1 to 3 mm. The computed area of the protein-rich region in the CT slice of the tumor was similar to the histological slice (Figure 4g, slope = 1.2, r^2^ = 0.92), and the mean CT number was -97±52 HU for a ∼1 mm^2^ circular ROI in the fat pad surrounding the tumor, compared to 68±44 HU for an ROI of the same area within the tumor (Figure 4h) (p<0.05). In all tumors, the CT number within the tumor was greater than in the surrounding fat pad (Figure 4i).

### CT Provides a Basis for Automated Tissue Segmentation

**Figure 5:**
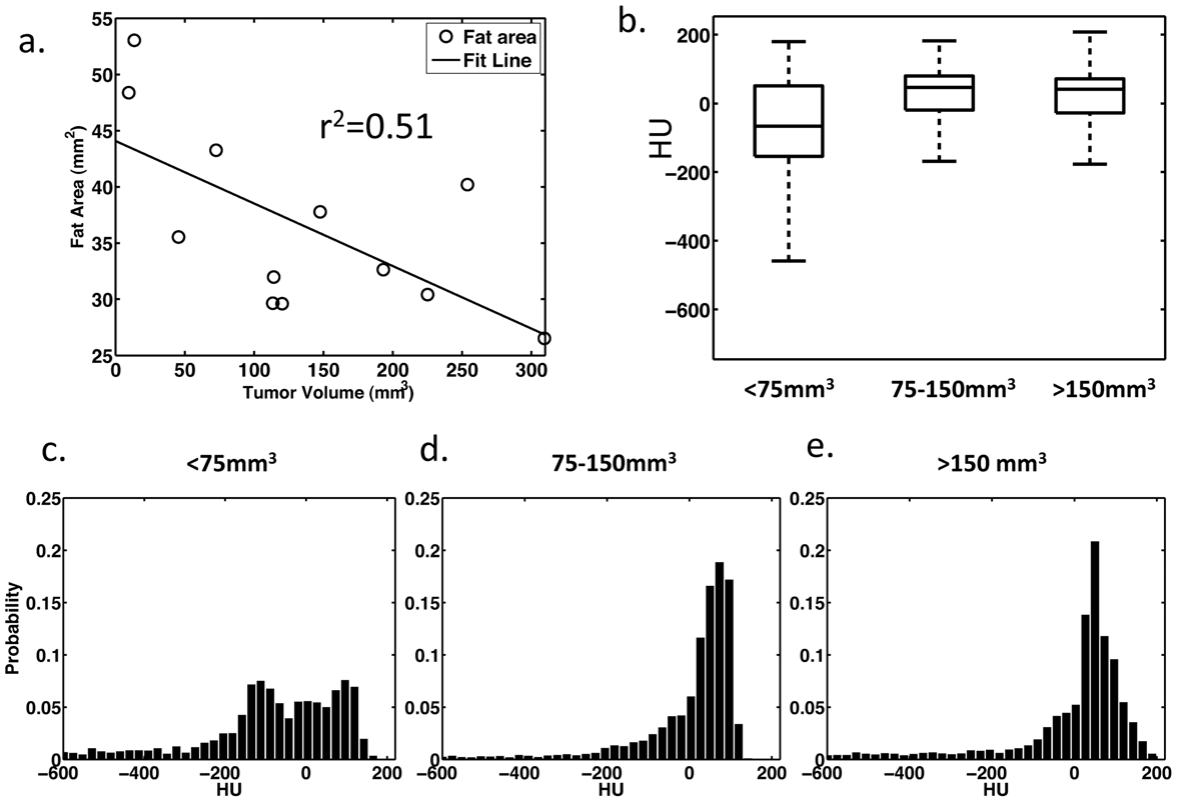
HU accurately predicts the tumor fat content. (a) Fat, as measured from histological slices, decreases with increasing tumor area (r^2^ = 0.51, slope = −0.3). (b) Box plot of average CT histograms show the fat shifted HU values in small tumors. (c–e) Average histograms of tumors in three tumor size groups show the presence of a tail in the range of HU associated with fat. Based on CT images and histology, fat content decreases with increasing tumor volume.

The area of fat in histology slices decreased with increasing tumor size (Figure 5a slope = -0.33, r^2^ = 0.51). Tumors with volumes less than 75 mm^3^ had a negative-shifted radiodensity on average, compared to tumors larger than 75 mm^3^, which had a higher probability of positively-shifted histograms (Figure 5b). The average percentage of fat measured from histology (%_fat_ = A_fat_/(A_fat_+A_tumor_)) for tumors <75 mm^3^, 75–150 mm^3^ and >150 mm^3^ was 75.2 ±11.8%, 48.4% ±3.3%, and 38.5 ± 8.2%, respectively, while CT quantitation yielded 65.3 ± 24.6%, 36.5 ±9.3%, and 31.6 ± 7.2%. Based on CT and histology, tumors with volumes less than 75 mm^3^ had a higher percentage of surrounding fat than larger tumors (p<0.05 for both measurement methods, t-test). Average histograms for the smallest tumors showed a greater count in the HU range below zero compared to larger tumors (Figure 5c–e).

### Fused CT/US imaging and tissue characterization is feasible *in vivo*

**Figure 6:**
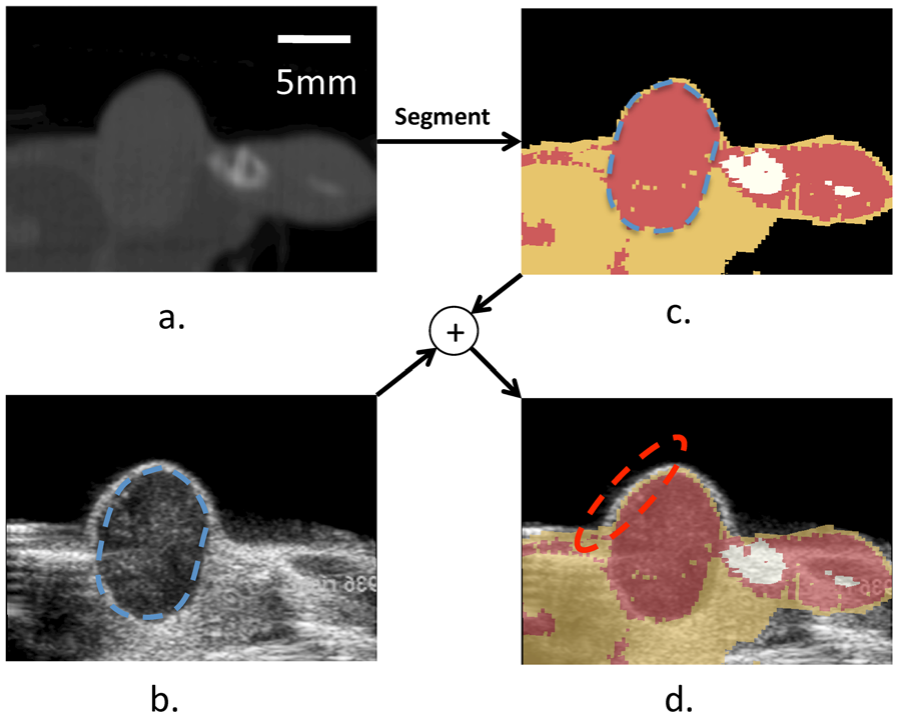
Combined CT/US characterizes tissue. (a) CT slice of a hindlimb tumor corresponding to the US slice in (b). The segmented CT slice is shown in (c) and overlaid on the US image in (d). The combined segmented CT/US image identifies fat proximal to and surrounding the tumor during ultrasonic imaging, with registration accuracy on the order of 1 mm. Nearby bone is obvious in the CT image (c) but not immediately visualized by US (b).

A real-time tissue-type overlay from CT was acquired *in vivo*, with a representative image from these studies shown in Figure 6a–d. The hypoechoic tumor embedded in the fat pad was imaged with US (Figure 6b, outlined in blue dotted lines), whereas the segmented CT images (Figure 6c) show a protein-rich region in the tumor core (red area) and the fatty regions surrounding the tumor (yellow area). The target registration error along the skin of the mouse (red ellipse) in the overlaid CT/US images (Figure 6d) was approximately 0.62 ± 0.13 mm.

## Discussion

We have synthesized a CT/US fusion capability that can combine images from generic clinical and pre-clinical ultrasound and CT scanners to provide real-time ultrasound imaging, informed by CT tissue characterization. Using 3D Slicer and OpenIGTLink, real-time US images can be overlaid on preacquired CT images, facilitating guidance of ultrasound imaging and therapy that is informed by tissue types. The combined CT/US system presented here has clinically relevant accuracy, and CT/US images of a living mouse demonstrate the feasibility of the fusion of images acquired from clinical scanners.

Most importantly, we found that even with tumors on the order of 1 cm and with minimal intervening tissue, CT could accurately characterize fat and bone surrounding the tumor, while ultrasound imaging facilitated recognition of the tumor boundaries. The amplitude of ultrasound echoes is altered by the intervening tissue and the thickness of tissues, such as the cortical bone in small animals. Artifacts, such as speckle and shadowing, also change the image amplitude depending on the location within the body and the acoustic path from the transducer to the tissue. Further, ultrasound cannot assess the distribution of tissue components at the sub-resolution (sub-beam dimensions) level, whereas CT can identify such components based on the distribution of Hounsfield units within a region.

With the acquisition parameters used here, voxel Hounsfield estimates from the dedicated breast scanner have a standard deviation of approximately 30 HU which allows for differentiation between fatty tissue (HU∼−120) and protein-rich tissue (HU>0) [21]. The use of cone-beam CT scanners raises concerns about spatially variant accuracy. Here, corrections for spatially variant noise were applied during reconstruction and our object is located near the axis of rotation where the highest accuracy is achieved [22]. Thus, in our study, CT identified regions of fat with sub-millimeter dimensions.

The high correlation between CT and histology for tumor area measurements suggests that CT can accurately define the tumor boundaries for therapeutic planning. Since reflection of ultrasound by bone can result in a local doubling of the thermal dose, accurate mapping of bony structures adjacent to or immediately distal to tumors is important and was successfully accomplished (Figure 6c). By fusing CT and US, bone in the acoustic beam can be identified in CT, and an acoustic path that avoids bone can be chosen.

### Fat Characterization Can Improve US-Based Thermometry and Beam Focusing

US estimates of temperature were determined by the product of a tissue-dependent constant and an apparent time shift detected by ultrasound. The tissue-dependent parameter *k_tissue_* describes the thermal expansion and sound speed of the tissue and has been reported to differ in magnitude and sign between fat and other tissues over the temperature range examined here. Therefore, without spatially-registered characterization of fat content, ultrasonic mapping of changes in temperature can be incorrect in magnitude and sign. The fused CT/US system developed here can characterize tissue in the acoustic beam and improve US thermometry by providing a map of thermal expansion parameters within the region of interest.

Further, due to the low sound speed of fat (∼1450 m/sec) compared to the assumed imaging sound speed of 1540 m/sec, the presence of fat in the acoustic path can de-focus the beam and displace the ultrasound focus away from the expected position [23]. The beam width of a 7.5 MHz transducer can double when subjected to an 8% error in sound speed compared to assumed sound speed [23]. For a therapeutic beam with a depth of focus on the order of 1–2 mm, unpredictable expansion and displacement of the acoustic focus would be significant. Moreover, while sound speed decreases in fat with increasing temperature, sound speed increases with temperature in most other tissues [16], [24]. Therefore, previous ultrasonic thermometry studies have created temperature maps by assuming a homogenous tissue and constant sound speed [14], [25].

Finally, the acoustic attenuation coefficient of fatty tissue is approximately 1 dB/(cm⋅MHz) lower than tumor and muscle; thus, temperature will increase more slowly in fat than in other tissues for a given thermal dose [26], [27]. Therefore, *a priori* predictions of the thermal dose that are required to produce a given temperature increase require an accurate estimate of local fat content. [13].

### Accuracy of combined CT/US is ∼1 mm

Registration accuracy on the millimeter scale is required to guide therapeutic US, but practical problems, such as tracking accuracy, sensor-transducer calibration, and mechanical contact of the transducer with the target decrease registration accuracy. The 6DOF EM sensors have a quoted accuracy of 1.1 mm and 0.6 degrees (95% confidence interval) [28]; however, the EM-detected static accuracy is below 1 mm within a subvolume of the tracked space that is sufficiently large for superficial tumors [29]. The single-point method of calibration used here is the most accurate method discussed in literature [30], and the problem of target deformation during US imaging was avoided by mechanically decoupling the animal from the US transducer. The resultant system achieved mm-scale accuracy that was confirmed by B-mode US images.

### Met-1 validated as model for breast tumor tissue characterization

The Met-1 tumor cells used in these studies provided a model for developing breast cancer. After transplantation into the fat pads, a growing tumor displaced the surrounding fat and infiltrated the tissue, increasing the mean HU values as compared to fat pads without a tumor. The protein-rich tumor embedded in the fat pad is a heterogeneous tissue with decreasing fat content as the tumor grows and provided a model to test the capabilities of CT tissue characterization.

### Study Limitations

For the first time, tissue characterization has been performed using fused, clinically-relevant CT and US images; however, there are some limitations that should be acknowledged. Temporal resolution of the image update is currently limited by the 30 Hz maximum frame rate output from our commercial US system. While the physical limitations of US allow for higher frame rates, a 30 Hz update rate is likely to be adequate for interventional applications. An alternative approach is to acquire raw radio frequency signals from a research-based ultrasonic system which operates at a higher frame rate [31]. Further, while phantom studies used a real-time overlay of CT and US, our *in vivo* study used a retrospective overlay due to logistics of the vivarium and US and CT scanners. However, *in vivo* tissue characterization is feasible (see Figure S1).

In addition, optimization of the HU values used in segmentation has not been pursued. While the HU limits we applied for segmentation have a physical basis and are similar to those found elsewhere in literature, methods to leverage *a priori* information and 3D morphological and region growth operations have been reported [32], and would likely increase the accuracy of segmentation. Finally, while the clinical CT scanners used here have a resolution of approximately 0.3 mm, which was sufficient to detect fat pads with thicknesses of 1–3 mm, the Siemens Inveon scanner, with a resolution of ∼0.1 mm, further improved fat pad tissue characterization and has been applied to characterize tumors based on *in vivo* imaging (see Figure S1).

The overall goal of this research was to create an imaging method that can accurately identify tissues in the acoustic beam path. Quantification of the sensitivity, specificity, and accuracy of such a system are important questions that are not fully addressed here. Future work will focus on quantifying the specificity, sensitivity, and accuracy of tissue characterization using fused CT/US.

### Summary and practical applications

In summary, we developed an open environment fused CT/US system with 1 mm resolution and applied this system to characterize bone, fat and tumor in a mouse model of developing cancer. Practical applications for this technology include acoustic therapy planning and enhancing US-based thermometry in heterogeneous tissue. In the future, the combined CT/US system will be integrated with ultrasound thermometry as described in [33], facilitating real-time control of hyperthermia as required for image-guided drug delivery and image-guided hyperthermia.

## Methods

### Animal Model and Imaging Procedures

The University of California at Davis Institutional Animal Care and Use Committee approved our study (protocol # 15864). Syngeneic Met-1 tumors grown within the mammary fat pad provided the model used here for tissue characterization and validation. Female FVB mice underwent bilateral transplantation of Met-1 tumor cells into the fourth fat pad. After tumor growth to a diameter of approximately 0.5 cm, seven mice with 12 tumors (two tumors did not develop) were imaged with CT after euthanasia using a dedicated breast CT [21]. All tumors were excised for H&E stained histology along the midline sagittal direction of the tumor.

Four female mice with Met-1 tumors within the mammary fat pad were imaged to test *in vivo* feasibility of fused CT/US. In these studies, the mice were imaged with tracked US prior to euthanasia and subsequently underwent CT imaging. The tracked US images were localized in the CT volume retrospectively using the tracking information and fiducial locations acquired during the US scan.

The feasibility of CT segmentation using *in vivo* images was demonstrated using a small animal Siemens Inveon CT (Erlangen, Germany) with a pixel dimension of 48.9 µm. Two mice with three tumors were imaged before euthanasia (Inveon: Bin 2, low magnification, Current/Voltage: 80 kVp/425 uA, 750 ms/projection, 180 projections). CT images and tissue-segmented images along with comparable histology are shown in Figure S1.

### Localization of Histological Slice in CT Images

Tumors were localized in the CT image stack by generating circular ROIs in multiple transverse images in the region of the hind limb using custom MATLAB software (MATLAB, Natwick, MA). The resultant ROI was sliced in the midline sagittal plane coincident with the histology slice, and the CT slice was cropped so only the tumor region was visible for comparison with histology, yielding a 2D CT image containing only the tumor and surrounding fat pad.

### CT Segmentation, Percentage Calculation, and ROI Selection

CT images were characterized as fat, protein-rich, or bone according to the values shown in Table 1 using 3D Slicer. The ranges were chosen based on observation of HU histograms of axial slices of tumors and are similar to those used by Borkan et al [34]. The area fat percentage within a 2D CT slice comparable to the histology slice was determined by computing the 2D area of fat after segmentation using HU in Table 1 normalized by the area of the tumor and surrounding fat (all measurements reported in Table 2). To measure the difference in radiodensity between the tumor and surrounding fat pad, we computed the mean HU of a circular ROI with an area of ∼1 mm^2^ from the 2D CT slice within the tumor and another ROI in the surrounding fat pad. Probability density functions of small (<75 mm^3^), medium (75–150 mm^3^), and large (>150 mm^3^) tumor groups were created from a HU histogram (128 bins) containing all pixels from the midline sagittal images of tumors from each group (MATLAB, Mathworks, Natick, MA). The resultant histogram was converted to a probability density function by dividing by the total area in the histogram.

**Table 2:** Area measurements of tumor region and surrounding fat pad from CT and Histology.

		CT			Histology		
	Tumor Volume (mm^3^)	Tumor Area (mm^2^)	Fat Area (mm^2^)	Percent Fat	Tumor Area (mm^2^)	Fat Area (mm^2^)	Percent Fat
	9.5	2.4	41.0	94.4	7.4	48.4	86.7
	13.5	16.9	33.3	66.4	10.5	53.0	83.5
**<75 mm^3^**	45.4	14.9	29.0	66.1	16.4	35.5	68.4
	74.9	33.3	17.4	34.3	26.3	43.3	62.2
	113.4	45.8	43.0	48.4	31.7	29.6	48.3
	114.1	38.7	20.1	34.2	30.1	32.0	51.5
**75**–**150 mm^3^**	120.2	46.7	28.0	37.5	37.8	29.6	43.9
	147.6	39.3	13.7	25.9	37.8	37.8	50.0
	193.2	52.7	35.3	40.1	40.1	32.6	44.8
	225.1	72.8	23.8	24.6	52.0	30.4	36.9
**>150 mm^3^**	253.9	62.0	33.1	34.8	49.3	40.2	44.9
	309.4	72.4	26.7	26.9	69.7	26.5	27.5

### ROI Selection and Volume Estimation from Histology

Histological images were used to validate the percentage of fat in the region surrounding the tumors and tumor volume. Two researchers blinded to tumor size selected ROIs containing either fat or tumor cells on histological images. Linear regression indicated similarity between the two measurement sets (fat area: r^2^ = 0.61, tumor area: r^2^ = 0.96). The mean of these two sets of measurements was used to minimize selection bias. The area of the fatty region was divided by the area of the combined tumor and fat regions. Tumor volume was estimated from histology by assuming an ellipsoidal shape and overlaying two radii of the tumor in the 2D slice (r_1_, r_2_), while the third radius (r_3_) was estimated by calculating the mean of the first two radii. Tumor volume was estimated by taking the volume of the ellipsoid with radii r_1_, r_2_, and r_3_ (measurements reported in Table 2).

### Statistical Methods

The area of tumors in the histological slice (as described in the methods) was compared to the tumor area computed from CT slices by linear regression with r^2^>0.5 considered significant. One-way ANOVA was used to test the ability for US to differentiate tissue types based on US image amplitude in tumor, fat pads, bone and muscle of 4 mice. Assuming a standard deviation of 10%, a change of 30% image amplitude can be detected between 4 tissues with a power of 80% and n = 4. Linear regression was performed to characterize general trends between tumor size and the area designated as fat by histology (r^2^>0.5 considered significant). Tumors were divided into small (n = 4, <75 mm^3^), medium (n = 4, 75–150 mm^3^), and large (n = 4, >150 mm^3^) size groups. With this sample size and assuming a standard deviation of 15%, the Student's t-test can detect differences of 35% in the regional percentage of fat with a power of 80%. We evaluated residuals of all measurements for normality and did not detect a substantial deviation from a normal distribution.

### Fused CT/US Imaging Hardware

An EM tracking system (NDI Aurora, Ontario, Canada) was used to detect the spatial location and orientation of sensors affixed to the US probe (as shown in Figure 1a). Within the complete cube volume (500 cm^3^), the tracking system's static position and orientation accuracy are 1.1 mm and 0.6 degrees, respectively, with a 95% confidence interval [28]. The 6 Degree of freedom (6DOF) sensors have improved static accuracy (less than 1 mm) when located within a 300 mm radial distance from the transmitter's origin [29]. Imaging and calibration were performed within this high performance subvolume. The video output of a clinical Sequoia ultrasound scanner (Siemens, Issaquah, WA, 15L8 transducer, 14 MHz) transmitted US images to a video capture card at 10 fps. The US scan plane was localized in the tracked volume and then mapped via fiducial registration to the CT volume. All images in the results section were acquired with a clinical dedicated breast CT [21] with a pixel dimension of 0.3 mm (breast CT scanner: X-ray Tube Current/Voltage: 7 mA/80 kVp, 530 projections).

### Tracked Imaging Software

The software component of our fused CT/US system is a plug-in created for 3D Slicer that imports the transducer position and corresponding US image and performs transformations for registration between CT and US, as described in the subsequent sections (plug-in source code, test data, and tutorial are available online: http://code.google.com/p/ct-us-openigtlink/) [35]. 3D Slicer provides the visualization interface for the combined image data. This software platform is open source, available on multiple operating systems, and has a modular architecture that allows for custom plug-ins to be created [35], [36], [37], [38]. The OpenIGTLink protocol is used to transfer US images and sensor position data to Slicer 3D [17]. Aurora tracking information is read using a device driver included in the Image Guided Surgery Toolkit (IGSTK), while images are captured with the Unicap library [18], [19].

### Image Registration

Following notation used in Prager et al., we use ^M^T_N_ to indicate a coordinate transform from an arbitrary coordinate system, **N**, into another system **M** and ^N^ **x** to represent a location in a coordinate system **N** (see coordinate systems in Figure 1a and Glossary of symbols, Table S1) [30]. Points in the scan plane (coordinate system **P**) must be transformed into the volume **C**, which is a 3D matrix of volume elements acquired by the CT scanner. The transformation of a pixel within the ultrasound image plane, ^P^ **x**, to the corresponding location within the CT volume, ^C^ **x**, can be described by:
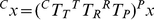
(1)


Each transformation consists of a translation and three Euler angle rotations. All transformations assume fixed axes and column vectors with rotations acting on objects and a rotation order of **z-y-x**. A general affine transformation between two coordinate systems can be achieved by:
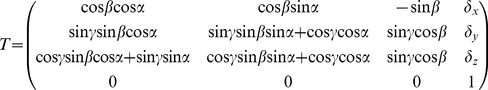
(2) with three rotations 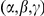 with respect to *z, y, x* and translations 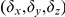 corresponding to *x*, *y*, and *z* respectively.

### Calibration Procedures

Accurate calculation of the orientation and translation between the transducer sensor and US image plane has been discussed previously, and many methods are outlined in [39]. Briefly, we used a single-point target method, where US acquired images of a fixed point from multiple angles while the tracking system returned position and orientation information for the attached sensor. In our calibration method, a cross-wire phantom was created with two pieces of 100-micron thread. The cross between the thread was assumed to be located at the origin of an arbitrary coordinate system, **A**, such that each image generated the following equation:
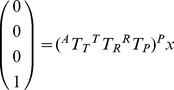
(3) where ^A^T_T_ represents the transformation from the EM transmitter into an arbitrary space centered at the line's midpoint. The point relative to the ultrasound image (**^P^x**) was calculated from manual measurements where the threads cross in the image (Figure S2a,b). The EM tracking system reported the associated sensor position and orientation (**^T^T_R_**). The resultant system of equations was solved for **^R^T_P_** using the Levenberg-Marquardt algorithm [40]. In solving Eq. 3, **^T^T_R_** is also optimized, but these values are discarded since **^T^T_R_** is measured from the tracking device during normal operation. An ensemble of observations was transformed according to Eq. 3 using measured values for **^T^T_R_**, and the average magnitude of the resultant vector indicated the residual error in the tracked ultrasound calibration.

### Registration Between Tracked Space and CT Image Space

The tracked volume and CT imaging stack were registered by selecting pairs of locations in the CT stack and the corresponding location in the tracked volume. Measurements in the tracked volume were made with a second EM sensor that was not attached to the ultrasound transducer. Given these paired measurements, the affine transformation between the CT stack and the tracked volume (**^T^T_C_**) was determined using Horn's method, as implemented in 3D Slicer [41]. Fiducial registration error is the average magnitude of the absolute difference between points transformed from the CT stack to the tracked volume. Target registration error in the cylindrical phantom was quantified by measuring the distance between circular ROIs centered on circular targets in fused images, and registration error in tumor images was quantified by measuring the mean difference in the skin boundary detected by CT versus US (ImageJ, NIH).

### Temporal Calibration

The 3D position, orientation, and image must be stored with the correct time-stamp to achieve accurate registration and reconstruction. In order to verify the accuracy of the timestamps, images of the base of a water bath were acquired along with the sensor location during the scans. The ultrasound technician moved the transducer in an oscillatory motion during the acquisition. The distance from the center of the transducer to the base of the water bath was measured from the US images by applying a threshold to generate binary images and counting the number of pixels from the center row of the image to the point on the line along the image row. The time lag between the sensor location and the distance to the bottom of the water bath measured from the US image was determined by taking the cross-correlation of both of the signals.

## Supporting Information

Figure S1 **Each row (a-c) shows histology, the comparable CT slice, and tissue segmented CT slice of three syngeneic mouse tumors imaged** ***in vivo*** **with the Siemens Inveon small animal scanner.** Images underwent a median filter with radius of 5 pixels prior to segmentation to reduce star artifacts from the CT scan.
(TIF)

Figure S2 **(a) The crosswire phantom is imaged at multiple angles by the transducer with attached 6DOF sensor. (b) The spatial location of the sensor and coordinates of the cross in the resultant image provide x^p^ and ^T^T_R_ for Eq. 3.**
(TIF)

Table S1 **Glossary of symbols.**
(DOCX)

## References

[1] Yoshizumi T, Nakamura T, Yamane M, Islam AH, Menju M (1999). Abdominal fat: standardized technique for measurement at CT.. Radiology.

[2] Wallace MJ, Kuo MD, Glaiberman C, Binkert CA, Orth RC (2009). Three-dimensional C-arm cone-beam CT: applications in the interventional suite.. J Vasc Interv Radiol.

[3] Ukimura O, Mitterberger M, Okihara K, Miki T, Pinggera GM (2008). Real-time virtual ultrasonographic radiofrequency ablation of renal cell carcinoma.. BJU International.

[4] Kitada T, Murakami T, Kuzushita N, Minamitani K, Nakajo K (2008). Effectiveness of real-time virtual sonography-guided radiofrequency ablation treatment for patients with hepatocellular carcinomas.. Hepatology Research.

[5] Kong G, Braun RD, Dewhirst MW (2000). Hyperthermia Enables Tumor-specific Nanoparticle Delivery: Effect of Particle Size.. Cancer Res.

[6] Lefor AT, Makohon S, Ackerman NB (1985). The effects of hyperthermia on vascular permeability in experimental liver metastasis.. Journal of Surgical Oncology.

[7] Brizel DM, Scully SP, Harrelson JM, Layfield LJ, Dodge RK (1996). Radiation Therapy and Hyperthermia Improve the Oxygenation of Human Soft Tissue Sarcomas.. Cancer Research.

[8] Song CW (1984). Effect of Local Hyperthermia on Blood Flow and Microenvironment: A Review.. Cancer Research.

[9] Dewey WC, Hopwood LE, Sapareto SA, Gerweck LE (1977). Cellular Responses to Combinations of Hyperthermia and Radiation.. Radiology.

[10] Hynynen K, Roemer R, Anhalt D, Johnson C, Xu ZX (1987). A scanned, focused, multiple transducer ultrasonic system for localized hyperthermia treatments.. International Journal of Hyperthermia.

[11] Dromi S, Frenkel V, Luk A, Traughber B, Angstadt M (2007). Pulsed-High Intensity Focused Ultrasound and Low Temperature, Sensitive Liposomes for Enhanced Targeted Drug Delivery and Antitumor Effect.. Clinical Cancer Research.

[12] Needham D, Anyarambhatla G, Kong G, Dewhirst MW (2000). A New Temperature-sensitive Liposome for Use with Mild Hyperthermia: Characterization and Testing in a Human Tumor Xenograft Model.. Cancer Res.

[13] Simon C, VanBaren P, Ebbini ES (1998). Two-dimensional temperature estimation using diagnostic ultrasound.. Ultrasonics, Ferroelectrics and Frequency Control, IEEE Transactions on.

[14] Seip R, VanBaren P, Cain CA, Ebbini ES (1996). Noninvasive real-time multipoint temperature control for ultrasound phased array treatments.. Ultrasonics, Ferroelectrics and Frequency Control, IEEE Transactions on.

[15] Rieke V, Butts Pauly K (2008). MR thermometry.. Journal of Magnetic Resonance Imaging.

[16] Hill C, Bamber J, Haar G (2004). Physical principles of medical ultrasonics: John Wiley & Sons Inc.

[17] Image Guided Therapy in Slicer3: Introduction to Navigation using OpenIGTLink.. http://www.slicer.org/slicerWiki/index.php/Modules:OpenIGTLinkIF-Documentation-3.6.

[18] unicap - The uniform API for image acquisition devices.. http://unicap-imaging.org/.

[19] Cleary K, Ibanez L, Ranjan S, Blake B (2004). IGSTK: a software toolkit for image-guided surgery applications.. International Congress Series.

[20] Fitzpatrick JM, West JB (2001). The distribution of target registration error in rigid-body point-based registration.. Medical Imaging, IEEE Transactions on.

[21] Boone JM, Nelson TR, Lindfors KK, Seibert JA (2001). Dedicated Breast CT: Radiation Dose and Image Quality Evaluation.. Radiology.

[22] Yang K, Kwan AL, Huang SY, Packard NJ, Boone JM (2008). Noise power properties of a cone-beam CT system for breast cancer detection.. Med Phys.

[23] Goss SA, Johnston RL, Dunn F (1978). Comprehensive compilation of empirical ultrasonic properties of mammalian tissues.. The Journal of the Acoustical Society of America.

[24] Abolhassani MD, Norouzy A, Takavar A, Ghanaati H (2007). Noninvasive Temperature Estimation Using Sonographic Digital Images.. J Ultrasound Med.

[25] Lai C, Kruse DE, Caskey CF, Stephens DN, Sutcliffe PL (2010). Noninvasive Thermometry Assisted by a Dual Function Ultrasound Transducer for Mild Hyperthermia.. IEEE Trans Ultrason Ferroelectr Freq Control.

[26] D'Astous FT, Foster FS (1986). Frequency dependence of ultrasound attenuation and backscatter in breast tissue.. Ultrasound in medicine & biology.

[27] Chivers RC, Hill CR (1975). Ultrasonic attenuation in human tissue.. Ultrasound in medicine & biology.

[28] Egorov V, Tsyuryupa S, Kanilo S, Kogit M, Sarvazyan A (2008). Soft tissue elastometer.. Med Eng Phys.

[29] Nafis C, Jensen V, Beauregard L, Anderson P (2006). Method for estimating dynamic EM tracking accuracy of surgical navigation tools..

[30] Prager R, Rohling R, Gee A, Berman L (1998). Rapid calibration for 3-D freehand ultrasound.. Ultrasound in medicine & biology.

[31] Pace DF, Gobbi DG, Wedlake C, Gumprecht J, Boisvert J (2009). An open-source real-time ultrasound reconstruction system for four-dimensional imaging of moving organs..

[32] Nelson TR, Cervino LI, Boone JM, Lindfors KK (2008). Classification of breast computed tomography data.. Medical Physics.

[33] Kruse DE, Chun-Yen L, Stephens DN, Sutcliffe P, Paoli EE (2010). Spatial and Temporal-Controlled Tissue Heating on a Modified Clinical Ultrasound Scanner for Generating Mild Hyperthermia in Tumors.. Biomedical Engineering, IEEE Transactions on.

[34] Borkan G, Gerzof S, Robbins A, Hults D, Silbert C (1982). Assessment of abdominal fat content by computed tomography.. The American Journal of Clinical Nutrition.

[35] Pieper S, Lorensen B, Schroeder W, Kikinis R (2006). The NA-MIC Kit: ITK, VTK, Pipelines, Grids and 3D Slicer as an Open Platform for the Medical Image Computing Community.. Proceedings of the 3rd IEEE International Symposium on Biomedical Imaging: From Nano to Macro.

[36] Pieper S, Halle M, Kikinis R (2004). 3D Slicer.. Proceedings of the 1st IEEE International Symposium on Biomedical Imaging: From Nano to Macro 2004.

[37] Gering D, Nabavi A, Kikinis R, Grimson W, Hata N (1999). An Integrated Visualization System for Surgical Planning and Guidance using Image Fusion and Interventional Imaging.. Int Conf Med Image Comput Comput Assist Interv.

[38] SlicerWeb http://www.slicer.org.

[39] Mercier L, Lang T, Lindseth F, Collins D (2005). A review of calibration techniques for freehand 3-D ultrasound systems.. Ultrasound in medicine & biology.

[40] More JJ (1977). Levenberg--Marquardt algorithm: implementation and theory..

[41] Horn BKP (1987). Closed-form solution of absolute orientation using unit quaternions.. J Opt Soc Am A.

