# Supplementary figures and images for: An Open Environment CT-US Fusion for Tissue Segmentation during Interventional Guidance
Source: PLoS One. 2011 Nov 23;6(11):e27372. doi: 10.1371/journal.pone.0027372 (PMC3223172; doi:10.1371/journal.pone.0027372)

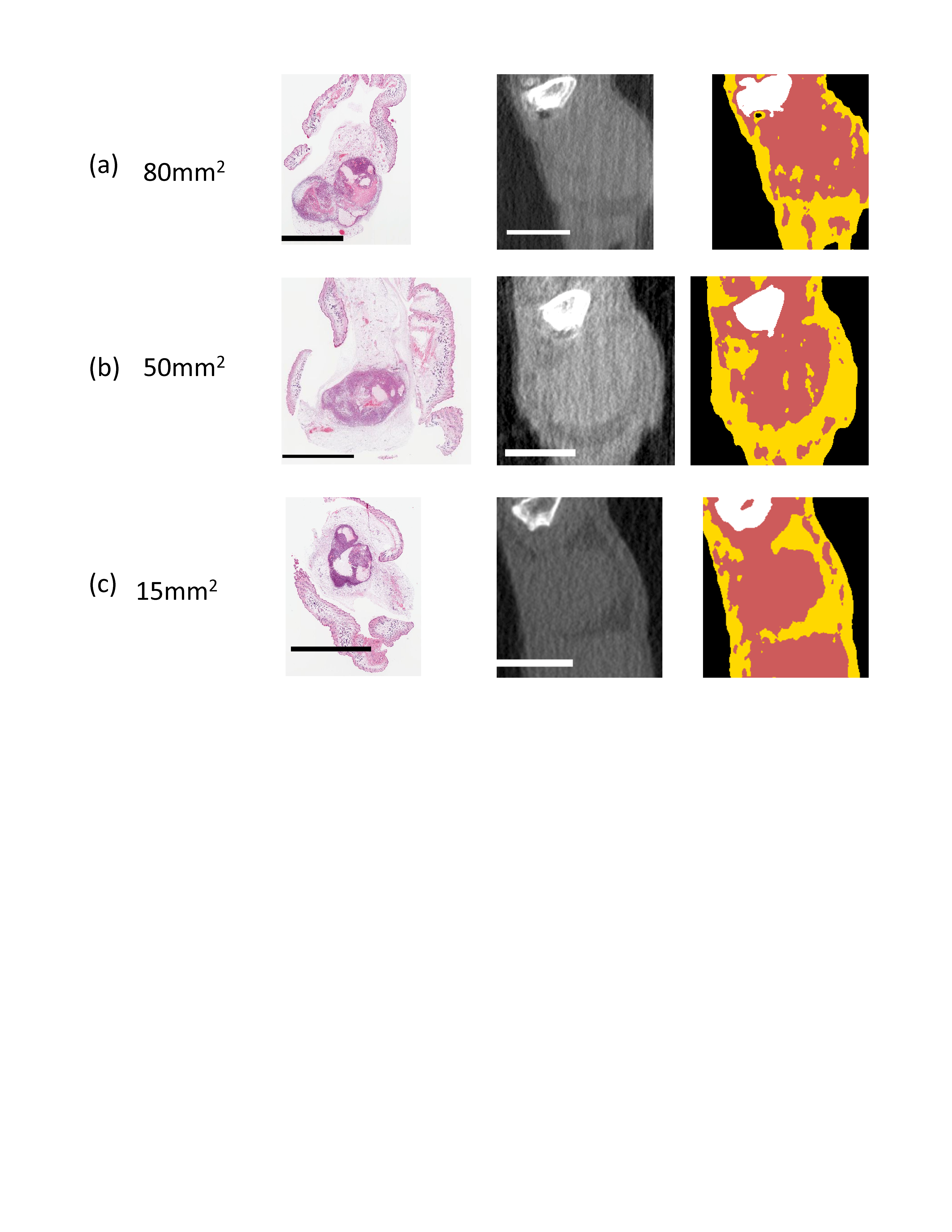

Supplement: Figure S1 — Each row (a-c) shows histology, the comparable CT slice, and tissue segmented CT slice of three syngeneic mouse tumors imaged in vivo with the Siemens Inveon small animal scanner. Images underwent a median filter with radius of 5 pixels prior to segmentation to reduce star artifacts from the CT scan. (TIF) [file pone.0027372.s001.tif]

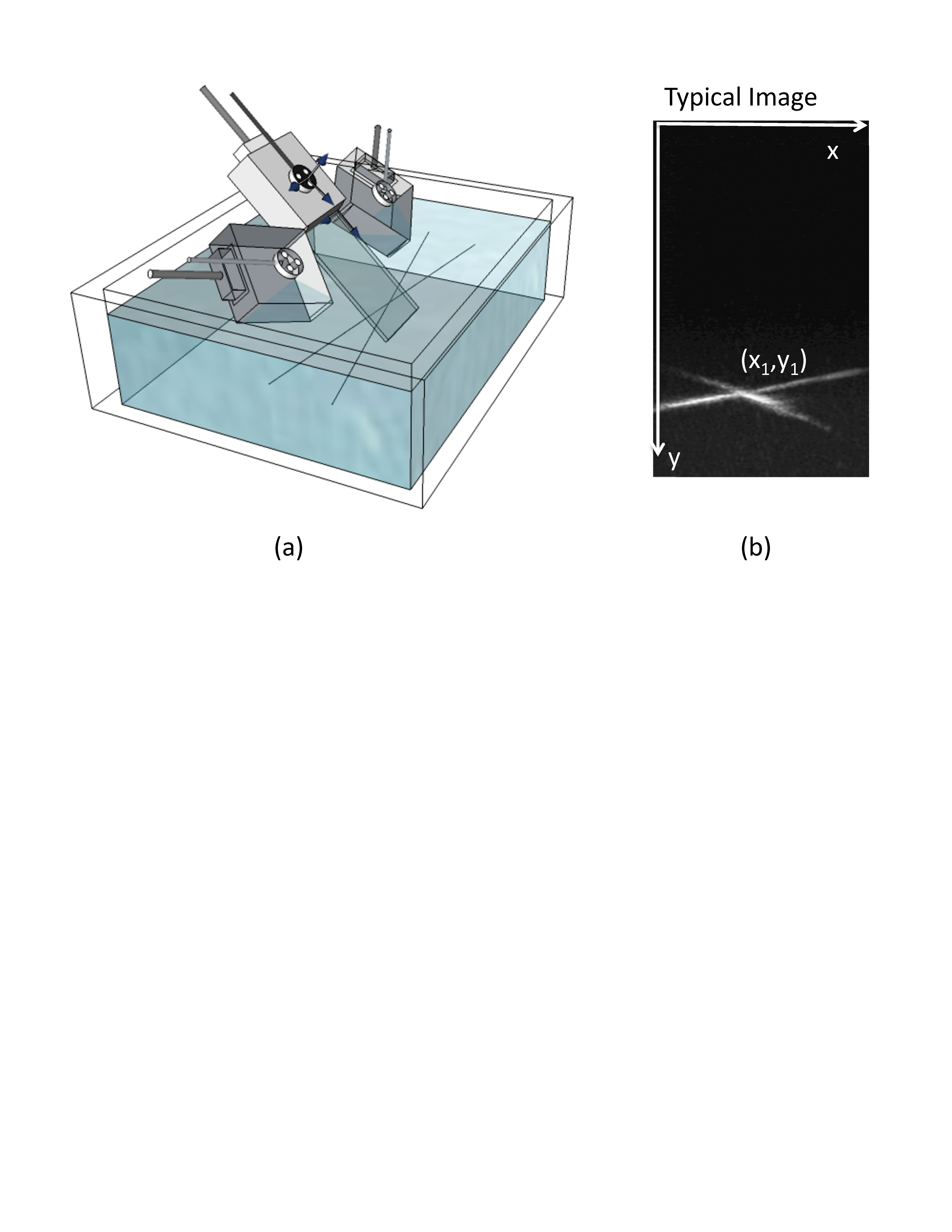

Supplement: Figure S2 — (a) The crosswire phantom is imaged at multiple angles by the transducer with attached 6DOF sensor. (b) The spatial location of the sensor and coordinates of the cross in the resultant image provide xp and TTR for Eq. 3. (TIF) [file pone.0027372.s002.tif]
