# Supplementary material for: An Open Environment CT-US Fusion for Tissue Segmentation during Interventional Guidance
Source: PLoS One. 2011 Nov 23;6(11):e27372. doi: 10.1371/journal.pone.0027372 (PMC3223172; doi:10.1371/journal.pone.0027372)
Supplement: Table S1 — Glossary of symbols. (DOCX) [file pone.0027372.s003.docx]

**Glossary of Symbols**

| ^P^**x** | Cartesian coordinate of the ultrasound plane |
| --- | --- |
| ^C^**x** | Cartesian coordinate in the CT image volume |
| **P** | 3D Coordinate system for ultrasound plane |
| **C** | 3D Coordinate system for CT volume |
| **R** | 3D Coordinate system for transducer sensor |
| **T** | 3D Coordinate system for EM transmitter |
| **A** | 3D Coordinate system centered at cross-wire for calibration |
| ^R^T_P_ | Rigid affine transformation from coordinate system P to R |
| ^T^T_R_ | Rigid affine transformation from coordinate system R to T |
| ^C^T_T_ | Rigid affine transformation from coordinate system T to C |
| ^A^T_T_ | Rigid affine transformation from coordinate system T to A |
| α | Rotation angle with respect to z-axis |
| β | Rotation angle with respect to y-axis |
| γ | Rotation angle with respect to x-axis |
| δ_X_ | Translation with respect to x-axis |
| δ_Y_ | Translation with respect to y-axis |
| δ_Z_ | Translation with respect to z-axis |
